# Characterizing adipocytokine-related signatures for prognosis prediction in prostate cancer

**DOI:** 10.3389/fcell.2024.1475980

**Published:** 2024-10-25

**Authors:** Shicheng Fan, Haolin Liu, Jian Hou, Guiying Zheng, Peng Gu, Xiaodong Liu

**Affiliations:** ^1^ Department of Urology, The First Affiliated Hospital of Kunming Medical University, Kunming, Yunnan, China; ^2^ Department of Urology, Institute of Urology, West China Hospital, Sichuan University, Chengdu, Sichuan, China

**Keywords:** prostate cancer, adipocytokine-related gene, prognosis, BNIP3L, immune infiltration

## Abstract

**Background:**

Prostate cancer (PCa) is a prevalent malignant tumor in males, with a significant incidence of biochemical recurrence (BCR) despite advancements in treatment. Adipose tissue surrounding the prostate, known as periprostatic adipose tissue (PPAT), contributes to PCa invasion through adipocytokine production. However, the relationship between adipocytokine-related genes and PCa prognosis remains understudied. This study was conducted to provide a theoretical basis and serve as a reference for the use of adipocytokine-related genes as prognostic markers in PCa.

**Methods:**

Transcriptome and survival data of PCa patients from The Cancer Genome Atlas (TCGA) database were analyzed. Differential gene expression analysis was conducted using the DESeq2 and limma packages. Prognostic genes were identified through univariate Cox regression and least absolute shrinkage and selection operator (LASSO) regression. A prognostic model was developed and validated utilizing receiver operating characteristic (ROC) and Kaplan-Meier (K-M) curves. Assessments of immune cell infiltration and drug sensitivity were also carried out. Subsequently, the function of BNIP3L gene in PCa was verified.

**Results:**

A total of 47 adipocytokine-related differentially expressed genes (DEGs) were identified. Five genes (PPARGC1A, APOE, BNIP3L, STEAP4, and C1QTNF3) were selected as prognostic markers. The prognostic model demonstrated significant predictive accuracy in both training and validation cohorts. Patients with higher risk scores exhibited poorer survival outcomes. Immune cell infiltration analysis revealed that the high-risk group had increased immune and ESTIMATE scores, while the low-risk group had higher tumor purity. *In vitro* experiments confirmed the suppressive effects of BNIP3L on PCa cell proliferation, migration, and invasion.

**Conclusion:**

The prognostic model independently predicts the survival of patients with PCa, aiding in prognostic prediction and therapeutic efficacy. It expands the study of adipocytokine-related genes in PCa, presenting novel targets for treatment.

## Introduction

Prostate cancer (PCa) is not the only most prevalent malignant tumor in males worldwide, but also has the second greatest incidence internationally and is the fifth largest cause of death from cancer (Sung et al., 2021). The prognosis of patients with PCa has greatly improved with advances in radical prostatectomy and radical radiation therapy (Faria et al., 2020).

However, approximately 20%–60% of these patients experience biochemical recurrence (BCR) within 10 years (Van den Broeck et al., 2019). Although androgen deprivation therapy is an effective treatment modality for advanced PCa, it can eventually lead to drug resistance and the development of more invasive and metastatic castration-resistant prostate cancer (CRPC) (Sridhar et al., 2014). PCa, a highly heterogeneous malignancy, warrants considerable attention due to its increasing incidence and mortality rates (Desai et al., 2022). Although early diagnosis can lead to favorable outcomes, patients with advanced CRPC often still have unsatisfactory prognoses despite the implementation of various comprehensive treatment strategies (Sandhu et al., 2021). The adipose tissue enveloping the prostate, known as periprostatic adipose tissue (PPAT), has been shown to contribute to the invasion of prostate cancer through the production of adipocytokines (Nassar et al., 2018). Although key adipocytokines known to play a role in prostate cancer include leptin, adiponectin, visfatin, fibroblast growth factor 21 (FGF21), and bone morphogenetic proteins (BMPs) (Adesunloye, 2021), there is a paucity of prognostic studies exploring the relationship between adipocytokine-related genes and PCa. Therefore, focusing on potential prognostic research into the relationship between PCa and adipocytokines is of paramount importance. Moreover, identifying more reliable and effective therapeutic targets is crucial for devising personalized treatment plans for patients with various forms of PCa.

Adipokines, which are biologically active molecules secreted by adipose tissue, play a significant role not only in metabolic regulation but also in the onset and progression of cancer (Calle and Kaaks, 2004). These molecules influence cellular behavior, including the proliferation, migration, and apoptosis of tumor cells, by binding to specific receptors and activating cellular signaling pathways.

The role of adipokines is particularly noteworthy in the context of PCa (Mistry et al., 2007). In individuals with obesity, the levels of inflammatory adipokines such as leptin, adiponectin, interleukin-6 (IL-6), heparin-binding epidermal growth factor-like growth factor (HB-EGF), and vascular endothelial growth factor (VEGF), which are secreted by adipose tissue, are elevated (Baillargeon and Rose, 2006). These factors may play an important role in cancer by modulating the activity of inflammatory cells and impacting the tumor microenvironment (Cancel et al., 2022). Moreover, the levels of adiponectin, an adipokine with anti-inflammatory and anti-atherogenic properties, are typically lower in individuals with obesity and diabetes. Several studies have found there to be a negative correlation between adiponectin levels and the risk of prostate cancer, suggesting its potential role in inhibiting cancer progression (Hu et al., 2019; Kashiwagi et al., 2024). However, the relationship between adipokines and PCa is complex, with different adipokines potentially playing opposing roles. For instance, leptin, which is primarily produced by adipose tissue, is elevated in individuals with obesity and has been associated with an increased risk of various cancers, including PCa (Xu et al., 2020). Additionally, adipokines may increase cancer risk by affecting the insulin and insulin-like growth factor (IGF) signaling pathways (Pollak, 2008).

Understanding the specific mechanisms by which adipokines act in PCa is crucial for developing new therapeutic strategies. Modulating the levels of adipokines or their signaling pathways may offer new avenues for PCa treatment. Furthermore, adipokines could potentially serve as biomarkers for PCa, aiding in its early diagnosis and prognostic assessment. An in-depth study of the biological functions of adipokines and their mechanisms of action in PCa is essential for fully understanding the complexity of cancer and devising new preventative and therapeutic strategies. However, the role of adipocytokines in the progression of PCa has not been fully investigated.

In this study, we performed differential gene expression analysis using PCa genomic data from The Cancer Genome Atlas (TCGA) and established a risk model according to univariate Cox analysis and least absolute shrinkage and selection operator (LASSO) regression to identify five adipocytokine-related genes as prognostic markers. The effectiveness of the risk model was validated using receiver operating characteristic (ROC) and Kaplan‒Meier (K–M) curves. Subsequently, a series of analyses were conducted on patients with varying risk scores to investigate their immune cell infiltration, potential for immunotherapy, and drug sensitivity. Finally, experiments with frozen human tissue samples confirmed the differential expression of five prognostic genes. *In vitro* experimental results validated the suppressive effects of BNIP3L on the proliferation, migration, and invasion of PCa cells. In conclusion, our study provides a novel prognostic biomarker for patients with PCa and offers valuable guidance for treatment decisions.

## Materials and methods

### Data acquisition

Transcriptome and survival data of patients with PCa were acquired from TCGA database (https://portal.gdc.cancer.gov/). A total of 481 primary tumor (PCa) and 51 normal samples in TCGA PCa dataset were selected for analysis; of these 481 PCa samples, 476 had effective disease-free survival (DFS) data. The GSE46602, GSE116918, and GSE69223 datasets were obtained from the Gene Expression Omnibus database (https://www.ncbi.nlm.nih.gov/geo/). GSE46602 has 36 PCa samples and 14 normal samples. The validation set GSE116918 has 248 samples with available DFS data. The GSE69223 dataset (15 PCa and 15 normal samples) was used for expression validation. In the GeneCards database (https://www.genecards.org/), there were 368 adipocytokine-related genes (Supplementary Table S1).

### Differential expression and functional enrichment analysis

Differentially expressed genes (DEGs) between 481 PCa and 51 normal samples were identified using the DESeq2 (v 1.32.0) package (Love et al., 2014) (*P* < 0.05 and |log2-fold change (FC)| > 0.5). Differential expression analysis was subsequently conducted on 36 PCa and 14 normal samples via the limma (v 3.44.2) package (Ritchie et al., 2015) in the GSE46602 dataset (*P* < 0.05 and |log2FC | > 0.5). Volcano plots and heatmaps were generated with the ggplot2 (v 3.3.2) (Ito and Murphy, 2013) and pheatmap (v 0.7.7) packages, respectively. To identify adipocytokine-related DEGs, we selected common genes that exhibited consistent expression patterns in both the TCGA and GSE46602 datasets and were known to be related to adipocytokines. Gene Ontology (GO) and Kyoto Encyclopedia of Genes and Genomes (KEGG) analyses of the adipocytokine-related DEGs were performed with the clusterProfiler (v 3.16.0) package (Wu et al., 2021) (*P* < 0.05).

### Screening of prognostic genes

TCGA dataset was randomly divided at a 7:3 ratio into a training set, consisting of 334 samples, and a testing set, comprising 142 samples. Subsequently, univariate Cox regression analysis was conducted on the identified adipocytokine-related DEGs to identify key genes (*P* < 0.05). Based on the key genes, LASSO regression was implemented using the glmnet (v 4.0-2) package (Friedman et al., 2010) (family = cox) to select prognostic genes. Furthermore, prognostic gene expression was validated across TCGA and GSE46602 datasets and depicted through boxplot visualization. Immunohistochemistry images from the Human Protein Atlas (HPA) database were subsequently utilized to further determine the protein expression levels of prognostic genes in normal (NC) and PCa tissues.

### Evaluation of the risk regression model

The risk score for each patient with PCa in both the training and validation cohorts was calculated based on the following formula:

$$\begin{array}{ll} Risk\;score= & 0.049\;*\left(C1QTNF3\right)+0.156*\left(APOE\right)\\ & +\left(–0.198\right)\;*\left(PPARGC1A\right)+\left(–0.192\right)\;*\left(BNIP3L\right)\\ & +\left(–0.149\right)\;*\left(STEAP4\right) \end{array}$$

Moreover, an optimal threshold was assessed by relying on the risk value to split patients with PCa into high- and low-risk groups. K‒M curves were plotted for the two groups. Receiver operating characteristic (ROC) curves were constructed for 1-, 3-, and 5-year survival predictions. Additionally, the relationships between clinical characteristics, such as age and TNM stage, and the risk model were statistically analyzed using the chi-square test, with significance set at *P* < 0.05. Finally, the same operation was performed on the GSE116918 dataset.

### Independent prognostic analysis

The clinicopathological characteristics from TCGA training set samples were subjected to univariate Cox regression analysis to investigate the risk model and its clinical significance. Following this, the factors identified as significantly different in the univariate Cox analysis were incorporated into a multivariate Cox analysis, accompanied by tests for the proportional hazards (PH) assumption. Additionally, a nomogram was developed to predict the 1-, 3-, and 5-year survival rates of patients with PCa. Moreover, the accuracy of the nomogram’s predictions was assessed using calibration curves. We then categorized various clinical traits within TCGA training set and analyzed any significant differences in risk values among these groups. Finally, the disparities between these groups were illustrated using boxplots for visual representation.

### Immune infiltration analysis

To investigate the immune infiltration status in PCa, first, ESTIMATE analysis was performed with the ESTIMATE (v 1.0.13) package, and the immune score, stromal score, ESTIMATEScore, and tumor purity of each sample were obtained. Furthermore, the single sample gene set enrichment analysis (ssGSEA) algorithm was used to determine the rank value of each gene based on the expression profile. The Wilcoxon test was then applied to compare the differences in immune cell infiltration abundance between the two groups (*P* < 0.05). Subsequently, Spearman correlation analysis was conducted to investigate the relationships between prognostic genes and differential immune cells (*P* < 0.05). To assess the relationship between the risk score and immune cells, the differences in the expression of 28 immune cells between the two groups were also analyzed using Wilcoxon analysis (*P* < 0.05).

### Analysis of immune function and drug sensitivity prediction

The expression levels of prognostic genes at immune checkpoints (ICs) were evaluated and are presented in the form of a boxplot. In parallel, the SubMap algorithm was implemented to predict the response of immune checkpoint receptors. Furthermore, the pRRophetic (v 0.5) package (Geeleher et al., 2014) was used to calculate a variety of drugs contained in the Genomics of Drug Sensitivity in Cancer database (www.cancerrxgene.org). Moreover, the half-maximal inhibitory concentration (IC50) values of different drugs for each sample were assessed. Concurrently, survival analysis was performed following the stratification of common drugs into high and low IC50 groups, which were determined based on their median IC50 values.

### Verification of the expression of prognostic genes

A total of five pairs of frozen tissue samples, including both cancerous (CA) and normal samples, were utilized in this study. Total RNA from the samples was then separated and purified via TRIzol (Invitrogen, Carlsbad, USA) in accordance with the instructions provided by the manufacturer. Then, a NanoPhotometer N50 was used to measure the concentration of the extracted RNA. Next, reverse transcription was conducted using a standard PCR apparatus and a SureScript First-strand cDNA Synthesis Kit (Servicebio; Wuhan; China). After that, 5–20 times as much ddH2O (RNase/DNase-free) was applied to dilute the cDNA before performing PCR amplification (CFX96 real-time quantitative PCR apparatus) under the following conditions: predenaturation for 1 min at 95°C, denaturation for 20 s at 95°C, annealing for 20 s at 55°C, and elongation for 30 s at 72°C. Supplementary Table S2 presents the primer sequences (Tsingke; Beijing; China). Using GAPDH as an internal control, gene expression was calculated using the $2^{-\Delta \Delta CT}$ method.

### Cell culture and transfection

Two human PCa cell lines, DU145 (Procell Pricella Biotechnology Co., Ltd.; Wuhan; China) and PC3 (Procell Pricella Biotechnology Co., Ltd.; Wuhan; China), were maintained in MEM (G4550; Solarbio; Beijing; China) and Ham’s F-12K (G4560; Solarbio; Beijing; China), respectively, supplemented with 10% FBS (G4201; Gibco; Thermo Fisher Scientific, Inc.) at 37°C in an atmosphere of 5% CO2. Subsequently, the cells were transfected according to the manufacturer’s protocol. BNIP3L (oe-BNIP3L) and its negative controls were purchased from Genomditech (Shanghai; China) Co., Ltd.

### Western blotting

Cells were washed with phosphate buffered saline (PBS) and then lysed with 300 μL of RIPA (P0013B; Beyotime; Shanghai; China) lysis buffer on ice for 15 min. The supernatant was collected after centrifugation, after which the protein concentrations were determined using the BCA assay. Forty micrograms of protein were loaded and subjected to electrophoresis, followed by transfer onto a polyvinylidene fluoride (PVDF) membrane. After blocking with 5% skim milk, the membrane was incubated with the primary antibody overnight at 4°C and then with the secondary antibody for 2 h at room temperature. Bands were detected using a chemiluminescence imaging system.

### CCK-8 assay

Cells were plated in a 96-well plate and cultured overnight. At various time points (0, 12, 24, 48 h), 10 μL of CCK-8 (BS350C; Biosharp; Hefei; China) solution was added, and the plates were incubated in the dark at 37°C for 2 h. The optical density (OD) of each well was measured at 450 nm using a microplate reader. Cell viability (%) was calculated as $\left(OD\;of\;control\;wells-OD\;of\;blank\;wells\right)/\;\left(OD\;of\;experimentalwells-OD\;of\;blank\;wells\right)\times 100\%$ .

### Transwell assay

Matrigel (C0372; Beyotime; Shanghai; China) was thawed overnight at 4°C, and all pipette tips, Transwell inserts, and 24-well plates used in the coating process were precooled at 4°C. Fifty microliters of Matrigel were added to each Transwell insert and allowed to polymerize at 37°C for 30 min. The cells were trypsinized with 0.25% trypsin (Gibco; Thermo Fisher Scientific, Inc.) and then harvested, after which 100 μL of cell suspension was seeded into Transwell inserts coated with Matrigel, followed by incubation in a cell culture incubator for 48 h. Two hundred microliters of 4% paraformaldehyde fixative were added, and the cells were fixed at room temperature 20°C for 30 min. After the Matrigel was carefully removed, the inserts were placed into wells containing 500 μL of crystal violet staining solution and stained at room temperature 20°C for 15 min. After rinsing with PBS (Gibco; Thermo Fisher Scientific, Inc.), the cells were observed for invasive capacity under a 100x microscope (Olympus; Tokyo; Japan) and photographed for documentation.

### Wound healing assay

Cells were trypsinized with 0.25% trypsin (Gibco; Thermo Fisher Scientific, Inc.) and collected, after which the cell concentration was adjusted to 2.5 × 10^4^ cells/mL. The cells were then plated into Ibidi wound-healing inserts, 100 μL of cell suspension was added to each side of the insert, and each group was tested in triplicate. After overnight culture until the confluence reached 95%, the plate was removed, the basic culture medium was aspirated once, and then the basic culture medium was added. Images were taken at 0 and 48 h for observation.

### Statistical analysis

Bioinformatic analysis were conducted in the R program. The experimental data were assessed using GraphPad Prism version 8 software. T-tests were utilized to compare the two data sets. Differences in different goups was significant at *p* < 0.05.

## Results

### Identification of DEGs related to adipocytokines and functional enrichment analysis in PCa

**FIGURE 1:**
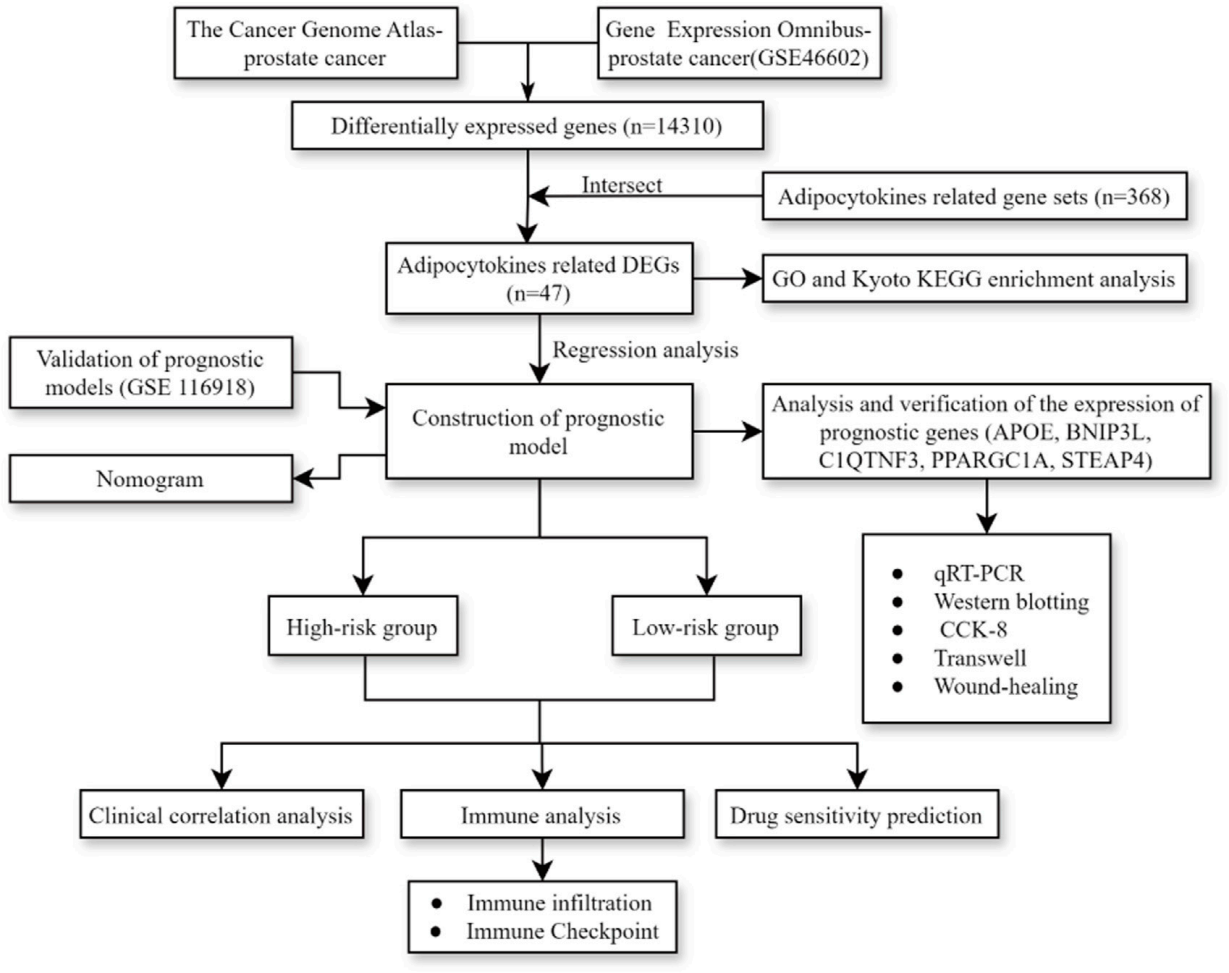
Process diagram of study.

**FIGURE 2:**
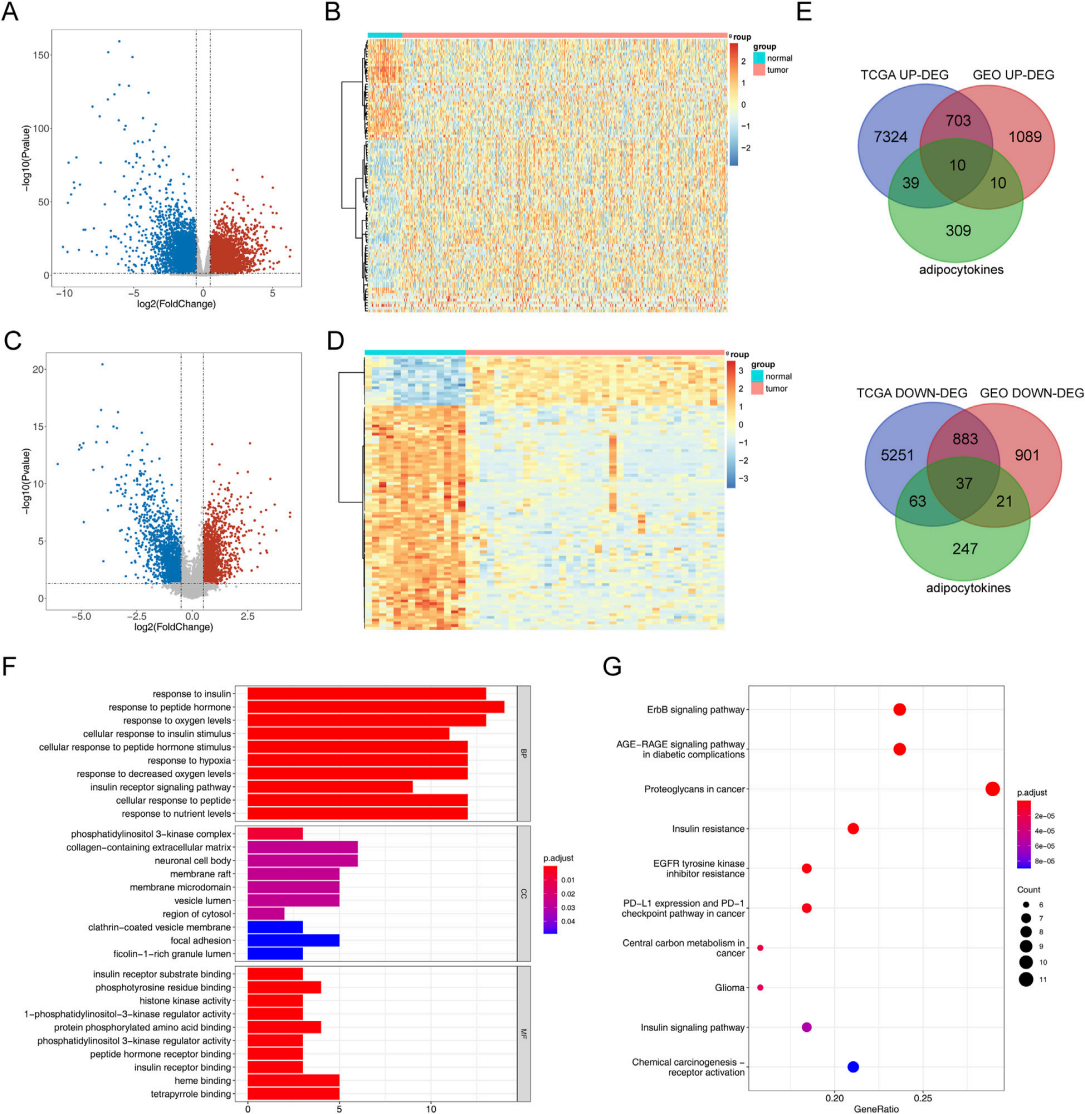
Analysis of differential expression and functional enrichment of adipocytokine-related genes from TCGA and GSE46602 datasets. **(A)** Volcano plot of DEGs1 in TCGA-PRAD. **(B)** Heatmap of DEGs1 in TCGA-PRAD cohort. **(C)** Volcano plot of DEGs2 in GSE46602. **(D)** Heatmap of DEGs2 in the GSE46602 dataset. **(E)** Venn diagrams of adipocytokine-related DEGs. **(F)** GO enrichment bar graph. **(G)** KEGG enrichment bar graph.

The process diagram of this study is presented in Figure 1. A total of 14,310 DEGs1 were screened in TCGA-PRAD dataset, including 8,076 upregulated genes and 6,234 downregulated genes (Figures 2A, B). A total of 3,654 DEGs2 were screened in the GSE46602 dataset, including 1,812 upregulated genes and 1,842 downregulated genes (Figures 2C, D). By taking the intersection of DEGs1 and DEGs2, 47 adipocytokine-related genes were obtained (Figure 2E). We also investigated the functions and related pathways of the identified adipocytokine-related DEGs. GO analysis revealed that the adipocytokine-related DEGs were related to the response to peptide hormones and insulin. In terms of cellular constituents, the primary associations were identified as being with the collagen-containing extracellular matrix and the neuronal cell body. From a molecular functional perspective, these DEGs are involved in heme binding and tetrapyrrole binding (Figure 2F; Supplementary Table S3). Moreover, KEGG analysis revealed that the adipocytokine-related DEGs participated in the ErbB signaling pathway and insulin resistance (Figure 2G; Supplementary Table S4).

### Construction and validation of an adipocytokine-related risk signature

**FIGURE 3:**
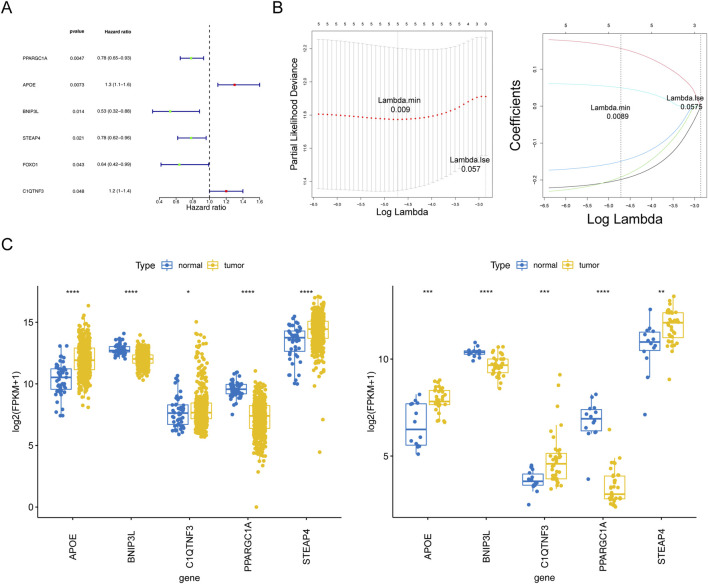
Statistical analysis of prognostic genes in prostate cancer. **(A)** Univariate Cox regression forest plot. **(B)** LASSO logistic regression coefficient penalty plot. **(C)** Boxplot of the prognostic genes in TCGA-PRAD and GSE46602 datasets. *P < 0.05; **P < 0.01; ***P < 0.001; ****P < 0.0001.

Based on the above 47 adipocytokine-related DEGs, we identified six key genes (PPARGC1A, APOE, BNIP3L, STEAP4, C1QTNF3, and FOXO1) through univariate analysis (Figure 3A; Supplementary Table S5). Then, five prognostic genes, namely, PPARGC1A, APOE, BNIP3L, STEAP4, and C1QTNF3, were identified through LASSO regression analysis (Figure 3B). Ultimately, a prognostic model for patients with PCa was established utilizing these five adipocytokine-related DEGs.

The expression trends of five genes in TCGA-PRAD and GSE46602 datasets were consistent and significant. As depicted in Figure 3C, compared to those in normal tissues, APOE, STEAP4, and C1QTNF3 expressions were upregulated in tumor tissues, whereas BNIP3L and PPARGC1A expressions were downregulated. Moreover, from the HPA database, we retrieved related immunohistochemistry images to further ascertain alterations in prognostic gene expression at the protein level. Notably, the HPA database does not provide information on the expression of STEAP4 and PP ARGC1A in PCa tissues. As demonstrated in Supplementary Figure S1, we discovered that the protein expression levels of BNIP3L were reduced, whereas those of APOE and C1QTNF3 were increased in patients with PCa compared to those in normal controls.

**FIGURE 4:**
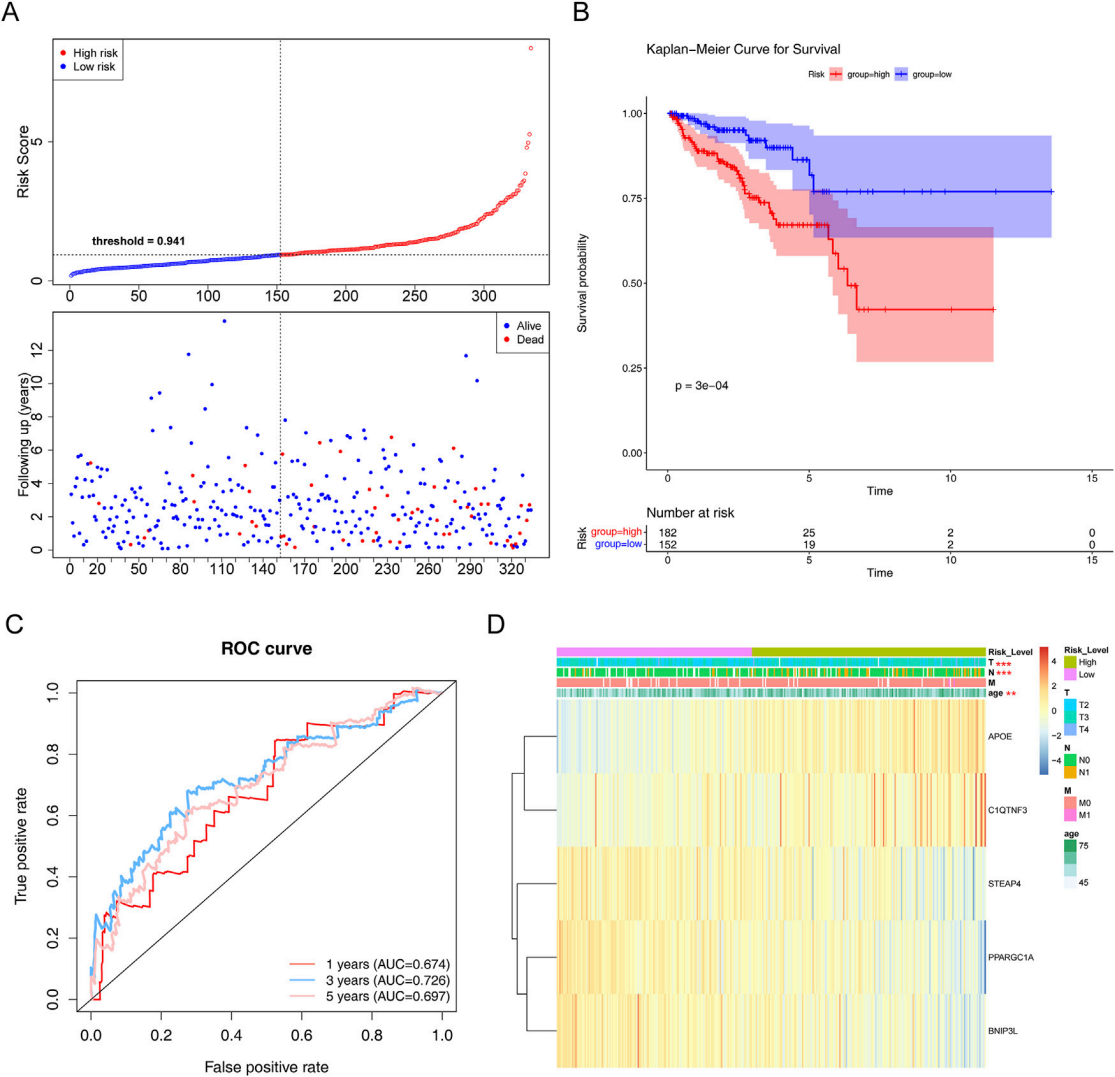
Evaluation of the risk regression model using the training set. **(A)** Risk curve for the two risk groups. **(B)** K–M survival curve. **(C)** ROC curves for the risk models. **(D)** Heatmap of the correlation between clinical features and the riskScore. **P < 0.01; ***P < 0.001.

To evaluate the prognostic value of the model, each patient’s risk value in the training set (334 samples) was computed by considering their gene expression levels. Patients with PCa were stratified into high- and low-risk groups based on a risk threshold value of 0.941. In the training set, there were notable differences in survival between the two risk groups (*P* < 0.05). The relationships between the risk score and survival time, survival status, and stratification are illustrated in Figure 4A. K–M survival curve analysis revealed a greater survival rate in the low-risk group (Figure 4B). Subsequently, the area under the ROC curve (AUC) values were calculated to evaluate the accuracy of the prognostic risk model in predicting 1-, 3-, and 5-year DFS, and were determined to be 0.674, 0.726, and 0.697, respectively. These results indicate that the constructed risk regression model had a certain predictive ability (Figure 4C). Moreover, we discovered that age, N (lymph node status), and T (tumor size) varied significantly across groups (Supplementary Table S6). The links between the risk factors, clinical traits and the expression of the five genes are shown in a heatmap (Figure 4D). In summary, these findings substantiate the robustness of our risk model in predicting the prognosis of patients with PCa.

**FIGURE 5:**
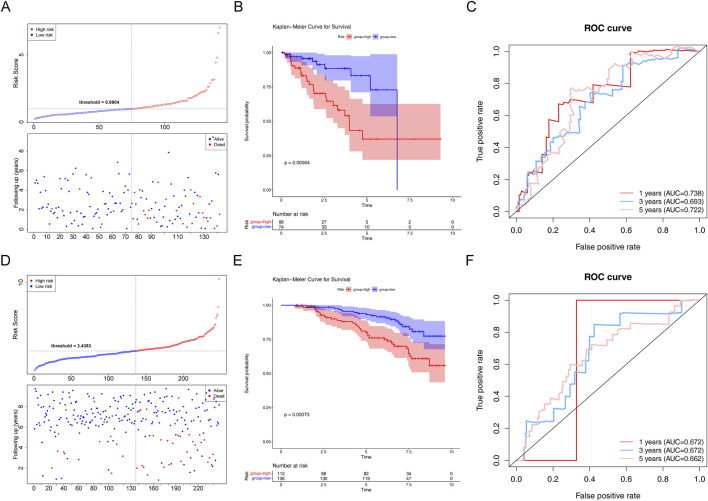
Assessment of the risk regression model using the testing and validation sets. **(A)** Risk curve for the two risk groups in the testing set. **(B)** K–M survival curve in the testing set. **(C)** ROC curves for the risk models in the testing set. **(D)** Risk curve for the two risk groups in the validation set. **(E)** K–M survival curve in the validation set. **(F)** ROC curves for the risk models in the validation set.

In the testing set, there was a substantial survival disparity between the two risk groups (*P* < 0.05) (Figures 5A, B). Undoubtedly, the high-risk group had a higher risk score. Moreover, we found that the area under the curve (AUC) was >0.65, proving that the risk regression model was effective (Figure 5C). Subsequently, in the GSE116918 validation set, the survival rates of the two risk groups differed considerably, with patients with PCa in the high-risk group having a shorter survival rate (Figures 5D, E). Through the ROC curves, we found that the area under the curve (AUC) was >0.65 for 1-, 3-, and 5-year survival, suggesting that the constructed risk regression model could be used as a prognostic model (Figure 5F).

### Construction of a nomogram and correlation analysis between the riskScore and clinical traits

The *P*-values of two factors (T stage and riskScore) were less than 0.05 according to the univariate Cox analysis (Supplementary Figure S2A; Table 1) and multivariate Cox analysis, suggesting that both have important significance in survival prognosis (Supplementary Figure S2B; Table 2). Thereafter, a nomogram for survival prediction in patients with PCa was constructed based on T stage and the riskScore (Supplementary Figure S2C). In addition, the slope was closest to 1 at 1 year, indicating that the prediction results were most accurate at this time point (Supplementary Figure S2D). Additionally, there were significant differences in the riskScore among the groups for age and M, N, and T stage. Specifically, patients aged 60 years or older with M1, N1, or T4 disease all had higher risk scores than their respective groups (Supplementary Figures S2E–H).

**TABLE 1:** Results from the univariate analysis.

Variable	coef	HR	HR.95L	HR.95H	*p*-value
Age	0.02653395	1.02688911	0.98299955	1.07273828	0.23381515
N	0.60573615	1.83260079	0.9815868	3.42142504	0.0572209
T	1.04772665	2.85116207	1.60841083	5.05413478	0.00033446
riskScore	0.52059275	1.68302497	1.31250617	2.15814076	4.07E-05

**TABLE 2:** Results of the multivariate analysis.

Id	coef	HR	HR.95L	HR.95H	*p*-value
T	0.89716895	2.45264969	1.38225842	4.351929	0.00216665
riskScore	0.44227114	1.55623765	1.20031886	2.01769354	0.00084371

### Immune cell infiltration landscape and drug sensitivity prediction

The tumor purity, immune scores, and ESTIMATE scores were notably different between the two groups. The high-risk group had increased immune and ESTIMATE scores, while the low-risk group had increased tumor purity (Supplementary Figures S3A–D). The different cell contents are shown in a heatmap (Supplementary Figure S3E). The results revealed a significant difference in the proportions of eight types of cells (activated CD8 T cells, CD56dim natural killer cells, effector memory CD4 T cells, eosinophils, myeloid-derived suppressor cells [MDSCs], immature dendritic cells, helper type 2 T [Th2] cells, and neutrophils) between the different groups (Supplementary Figure S3F). Additionally, there was a significant negative correlation between STEAP4 and CD56dim natural killer cells (|cor| = −0.377649058, *P* < 0.01) and between STEAP4 and central memory CD4 T cells (|cor| = −0.356259969, *P* < 0.01). There was a strong positive correlation between natural killer cells and PPARGC1A (cor = 0.524079876, *P* < 0.01) (Supplementary Figure S3G; Supplementary Table S7). Immunological checkpoint molecules are necessary for immunological function. Therefore, we studied the potential correlation between PCa and immune checkpoint molecule expression. The immune checkpoint molecules of seven genes (ICOS, TIGIT, CD27, HAVCR2, PDCD1, LAG3, and IDO1) were significantly different between the two risk groups (Supplementary Figure S4A). Anti-PD1 therapy was anticipated to have a greater impact on the high-risk group, whereas the low-risk group was predicted to be more susceptible to anti-CTLA4 therapy (Supplementary Figure S4B). Survival increased in the group treated with docetaxel at a high IC50 (Supplementary Figure S4C). These insights have significant implications for the therapeutic management of PCa.

### BNIP3L is decreased in cancer tissue samples

**FIGURE 6:**
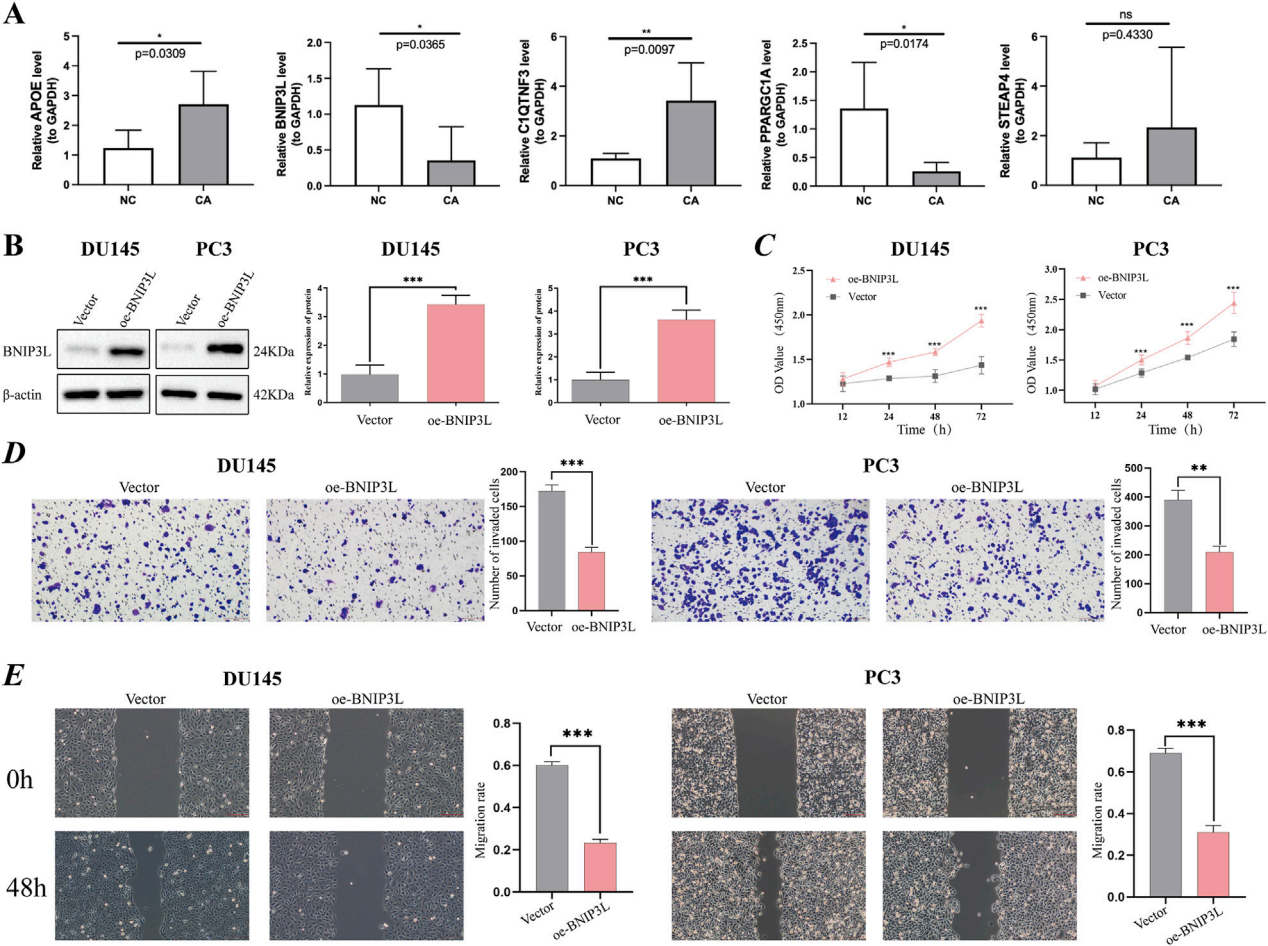
Verification of potential prognostic genes. **(A)** Relative mRNA expression of prognostic genes (APOE, BNIP3L, C1QTNF3, PPARGC1A, and STEAP4) in PCa tissue and paracancerous tissues. **(B)** The overexpression efficiency of the BNIP3L gene in DU145 and PC3 cells was assessed using Western blotting. **(C)** CCK-8 cell proliferation assay after BNIP3L overexpression in the DU145 and PC3 cell lines. The invasive and migratory abilities of DU145 and PC3 cells overexpressing the BNIP3L gene were investigated using Transwell assays **(D)** and wound healing assays **(E)**. *P < 0.05; **P < 0.01; ***P < 0.001; ns: Not significant.

Ultimately, our objective was to identify genes that play a role in prognosis and have not yet been extensively investigated in the context of PCa research. According to the RT‒qPCR verification results using five pairs of frozen samples, APOE and C1QTNF3 were upregulated in CA samples, and BNIP3L and PPARGC1A were downregulated in CA samples, consistent with the above analysis (Figure 6A).

### Overexpressing BNIP3L in prostate cancer cells decreases viability and invasion

Given the extensive research conducted on PPARGC1A, APOE, STEAP4, and C1QTNF3 in PCa, there remains a gap in fully elucidating the association between BNIP3L and PCa. One of the primary objectives of our study is to identify biomarkers that have not been thoroughly explored in the context of PCa. To this end, we have undertaken *in vitro* assays aimed at investigating the potential role of BNIP3L in the progression of PCa. Initially, BNIP3L was overexpressed in the DU145 and PC3 cell lines, and BNIP3L overexpression was confirmed through WB (Figure 6B). Subsequently, we assessed the impact of BNIP3L upregulation on the proliferation of PCa cells using a CCK-8 assay. The CCK-8 assay demonstrated that BNIP3L overexpression reduced the proliferative capacity of DU145 and PC3 cells compared to the empty vector control (Figure 6C). Transwell assays also demonstrated that overexpression of BNIP3L significantly inhibited the invasive capacity of DU145 and PC3 cells (Figure 6D), while wound healing assays indicated that upregulation of BNIP3L significantly delayed wound healing (Figure 6E). These findings suggest that BNIP3L inhibits the proliferation, migration, and invasion of PCa cells, suggesting that BNIP3L is a potential target for PCa therapy.

## Discussion

In this study, we identified 47 adipocytokine-related DEGs and used LASSO regression to demonstrate that PPARGC1A, APOE, BNIP3L, STEAP4, and C1QTNF3 were associated with patient survival, thereby developing a validated prognostic model. According to our ROC and K–M analyses, our findings indicate that the model is independent of clinical factors and holds promise as a prognostic factor for PCa. External validation using the GSE46602 dataset further substantiated the effectiveness of our prognostic model in differentiating patients with high- and low-risk PCa. This study demonstrates that the five prognostic signatures related to adipocytokines, identified using machine learning algorithms, have significant independent implications for predicting the prognosis of patients with PCa. Compelling evidence suggests that PPARGC1A may play a dual role in promoting and inhibiting the development of prostate tumors under certain conditions (Zheng et al., 2022). Recent studies (Bancaro et al., 2023) have indicated that APOE secreted by prostate tumor cells binds to triggering receptor expressed on myeloid cells 2 (TREM2) on neutrophils, thereby promoting their senescence. Both APOE and TREM2 exhibit increased expression in PCa and are associated with poor prognosis. Reports indicate (Li et al., 2021) that silencing STEAP4 leads to the activation of the cGMP-PKG signaling pathway, which inhibits lipopolysaccharide-induced proliferation in PCa cells. Investigations into the mechanism by which C1QTNF3 stimulates prostate cell proliferation have shown that it exerts antiapoptotic effects through the protein kinase C (PKC) signaling pathway (Hou et al., 2015). According to the RT‒qPCR results, APOE and C1QTNF3 were upregulated, while BNIP3L and PPARGC1A were downregulated in CA samples, which is generally consistent with the findings of the aforementioned studies. Notably, our RT-qPCR results showed no significant difference in STEAP4 expression between CA samples and normal samples, which is inconsistent with TCGA database and the aforementioned studies, possibly due to the heterogeneity of the tumors.

BNIP3L is a Bcl-2 family protein (Matsushima et al., 1998) that is essential for both apoptosis and mitophagy (Diwan et al., 2009), the latter being a process that degrades mitochondria and can potentially limit tumor growth (Chang et al., 2017). Dysregulation of mitophagy-related proteins, such as FUNDC1, Parkin, BNIP3, BNIP3L/NIX, and p62/SQSTM1, has been shown to be correlated with cancer (Drake et al., 2017). Reports indicate that BNIP3L knockout can prevent p53-dependent apoptosis under hypoxic conditions, thereby promoting tumor development (Fei et al., 2004). BNIP3L recruits TR3 to mitochondria, leading to autophagic cell death in melanoma (Wang et al., 2014). Furthermore, the oncogenic microRNA mir-30d facilitates tumor progression and adversely affects the expression of autophagy-related genes, including BNIP3L (Yang et al., 2013). Ultimately, another study has demonstrated that the endogenous degradation of BNIP3L confers survival to Ewing’s sarcoma cells (Gallegos et al., 2019). These observations suggest that BNIP3L-mediated autophagy/ferroptosis accelerates the demise of cancer cells. However, research on BNIP3L in PCa has predominantly focused on bioinformatics analysis (Liu et al., 2008; Cheng et al., 2012; Liu et al., 2020; Wen et al., 2022; Watanabe et al., 2024), and there is scant evidence from *in vitro* or *in vivo* experimental investigations. Consequently, we selected BNIP3L for subsequent experimental validation. Both TCGA database analysis and the RT-qPCR results indicate that BNIP3L is underexpressed in PCa tissues. We then explored whether enhancing its expression could inhibit the progression of PCa cells by conducting *in vitro* experiments, including WB, CCK-8, Transwell, and wound healing assays. The results of these assays demonstrated that overexpression of BNIP3L can suppress the proliferation, migration, and invasion of PCa cells. Although our *in vitro* experimental results are consistent with the aforementioned studies and are likely mediated through the regulation of mitophagy, the specific regulatory mechanisms require further investigation. Nevertheless, BNIP3L remains a promising prognostic marker and therapeutic target related to PCa and adipocytokines.

Overall, this study systematically analyzed the role of adipocytokine-related genes in PCa and successfully constructed and validated a prognostic model with potential clinical application prospects, identifying new molecular markers and therapeutic targets for precision medicine in PCa. Future research should focus on exploring the specific mechanisms of these genes in the development of PCa, as well as how to translate these findings into practical clinical applications. Our study, while providing notable results, still has some limitations. This study was retrospective, and our model’s ROC analysis showed only moderate accuracy in predicting PCa prognosis. Furthermore, the small sample size may have affected the outcomes. Additionally, our cellular experiments may have been inadequate because we only preliminarily investigated the functional phenotypes of BNIP3L in PCa cells; therefore, future *in vivo* experiments should be undertaken for further validation. In summary, the utilization of adipocytokine-related gene signatures for independent prognostic prediction in patients with PCa may provide valuable insights for therapeutic targets in patient management.

## Data Availability

The original contributions presented in the study are included in the article/Supplementary Material, further inquiries can be directed to the corresponding authors.
