# Supplementary figures and images for: Characterizing adipocytokine-related signatures for prognosis prediction in prostate cancer
Source: Front Cell Dev Biol. 2024 Oct 25;12:1475980. doi: 10.3389/fcell.2024.1475980 (PMC11544632; doi:10.3389/fcell.2024.1475980)

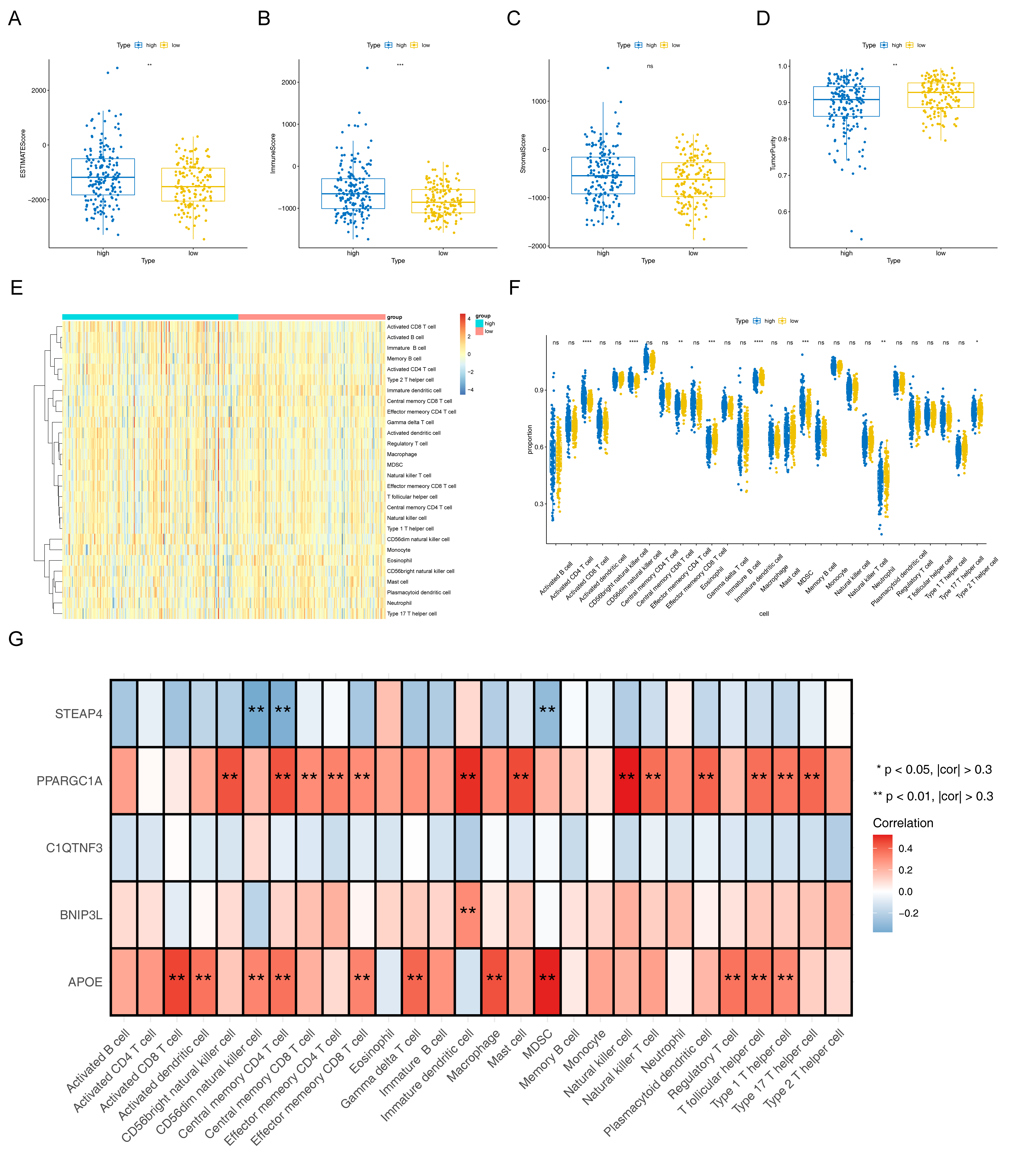

Supplement: Supplementary file 3 [file Image3.tif]

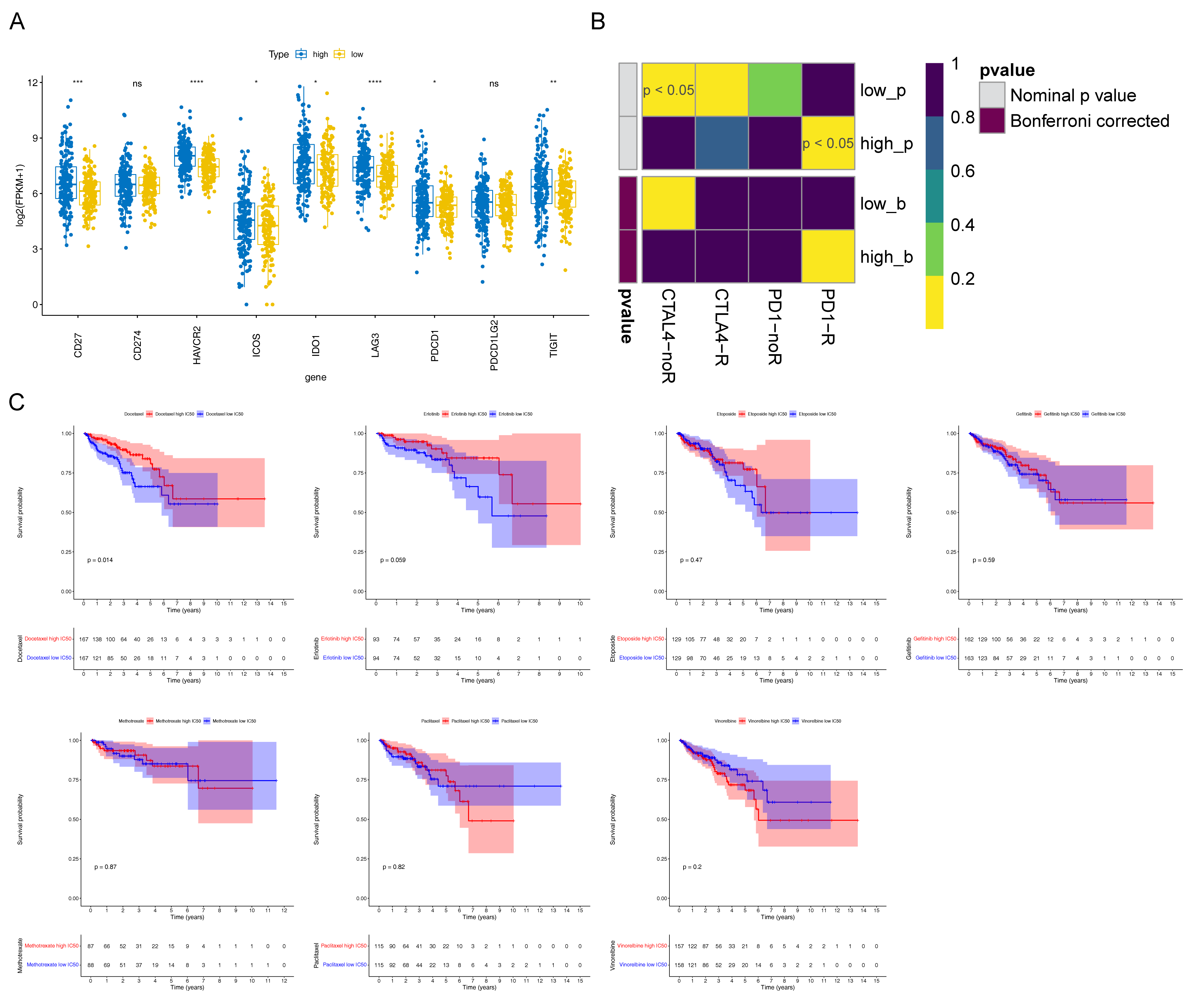

Supplement: Supplementary file 4 [file Image4.tif]

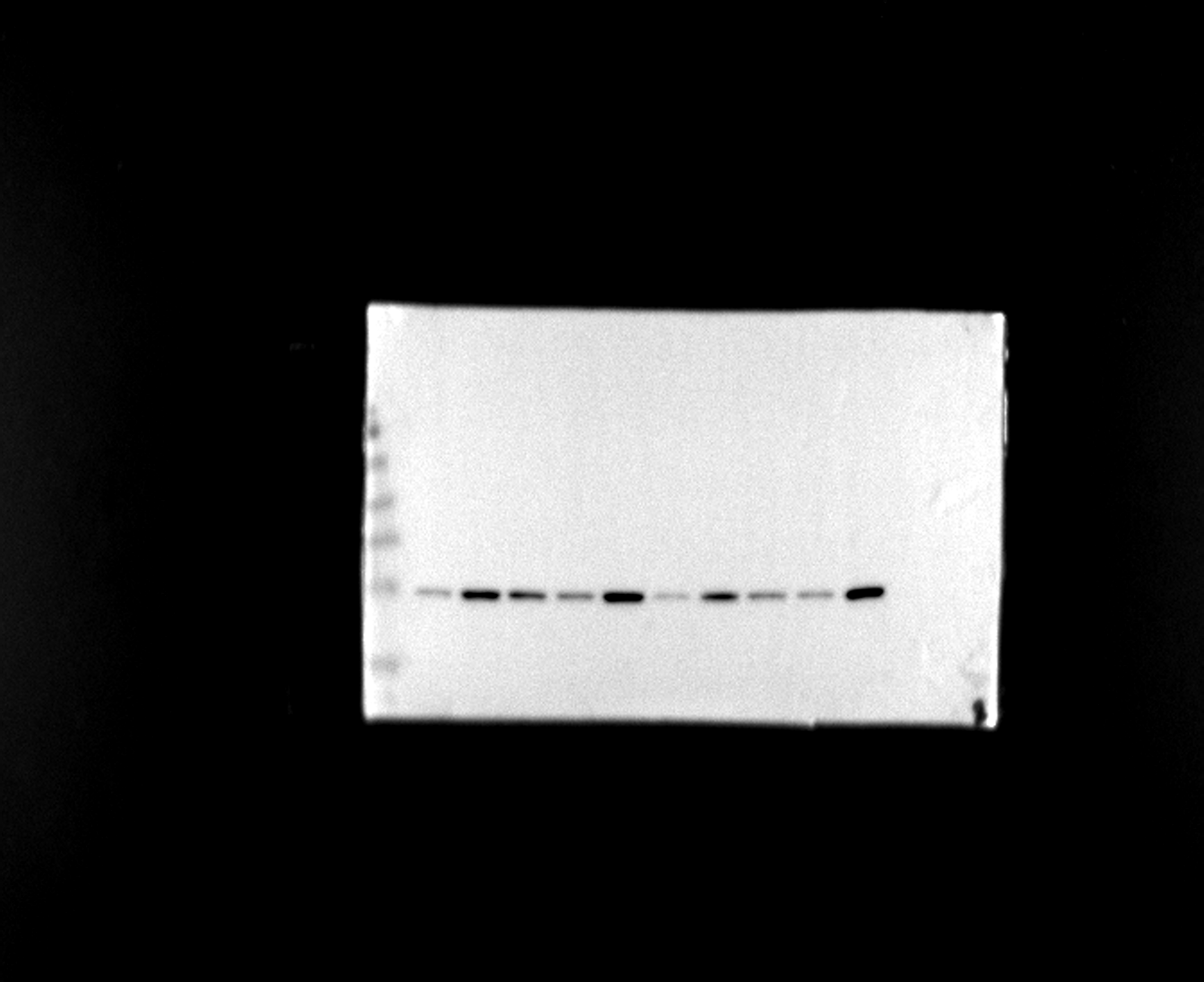

Supplement: Supplementary file 5 [file DataSheet1.zip › western blot images/oeBNIP3L-DU145.Tif]

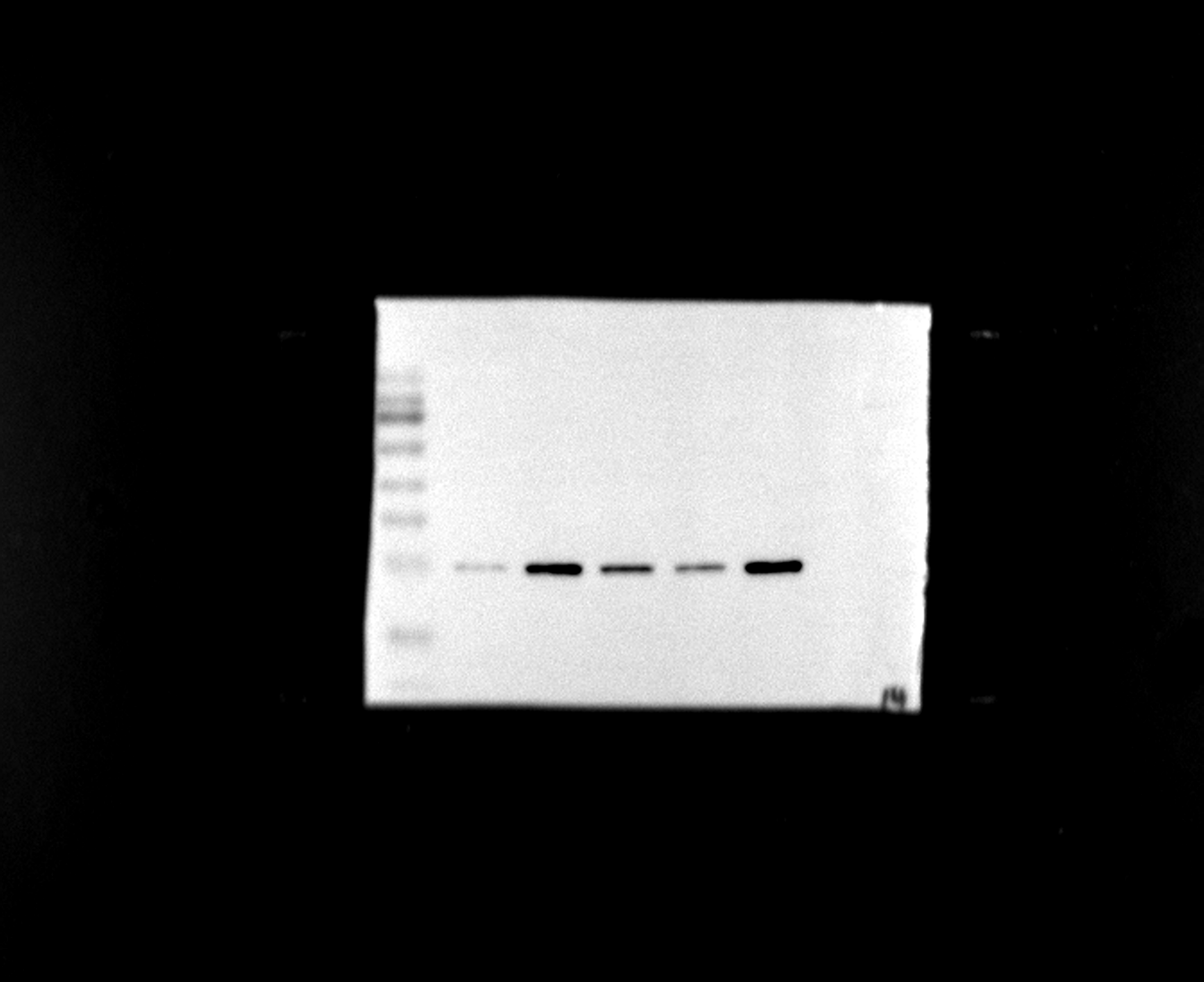

Supplement: Supplementary file 5 [file DataSheet1.zip › western blot images/oeBNIP3L-PC3.Tif]

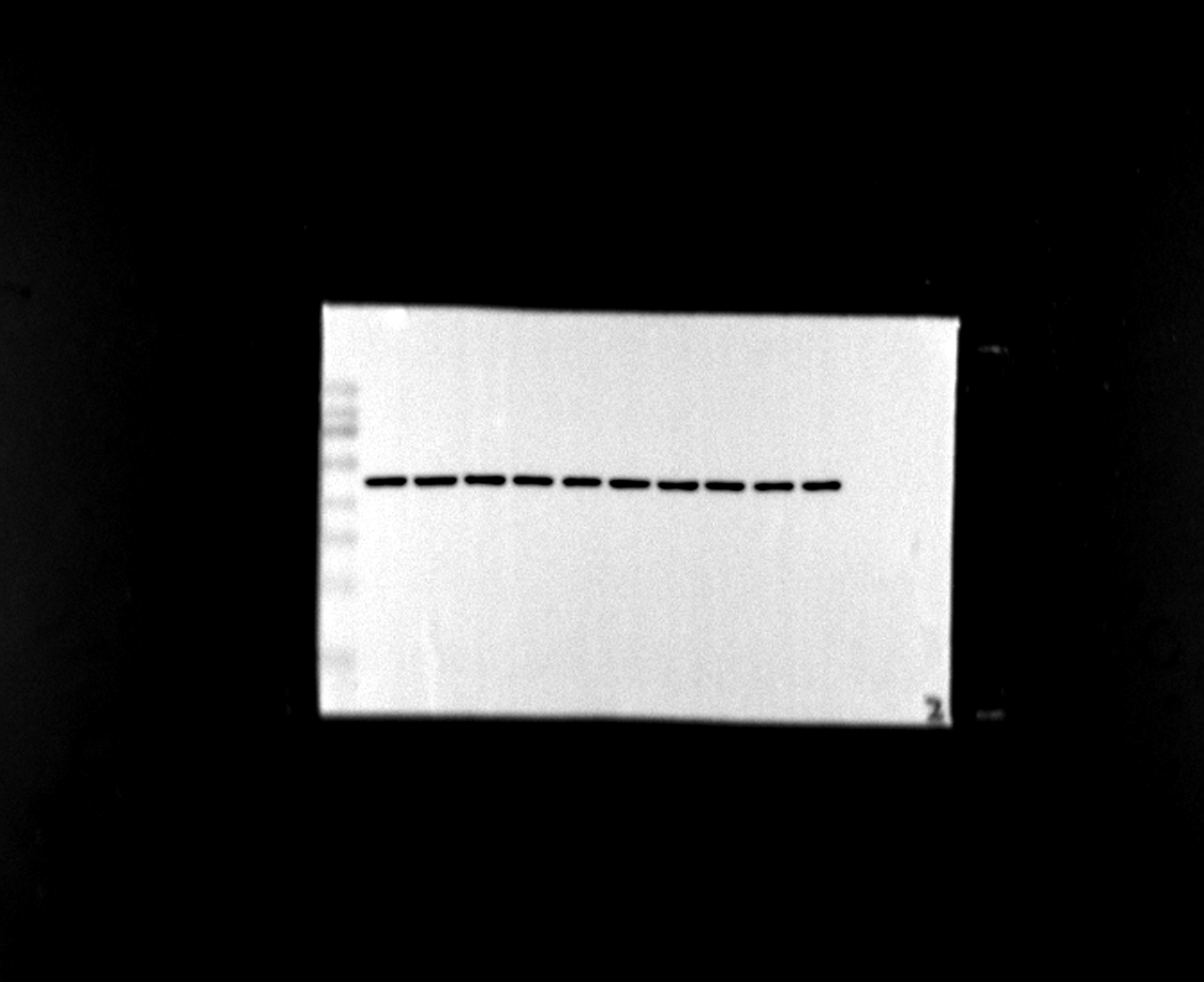

Supplement: Supplementary file 5 [file DataSheet1.zip › western blot images/β-actin-DU145.Tif]

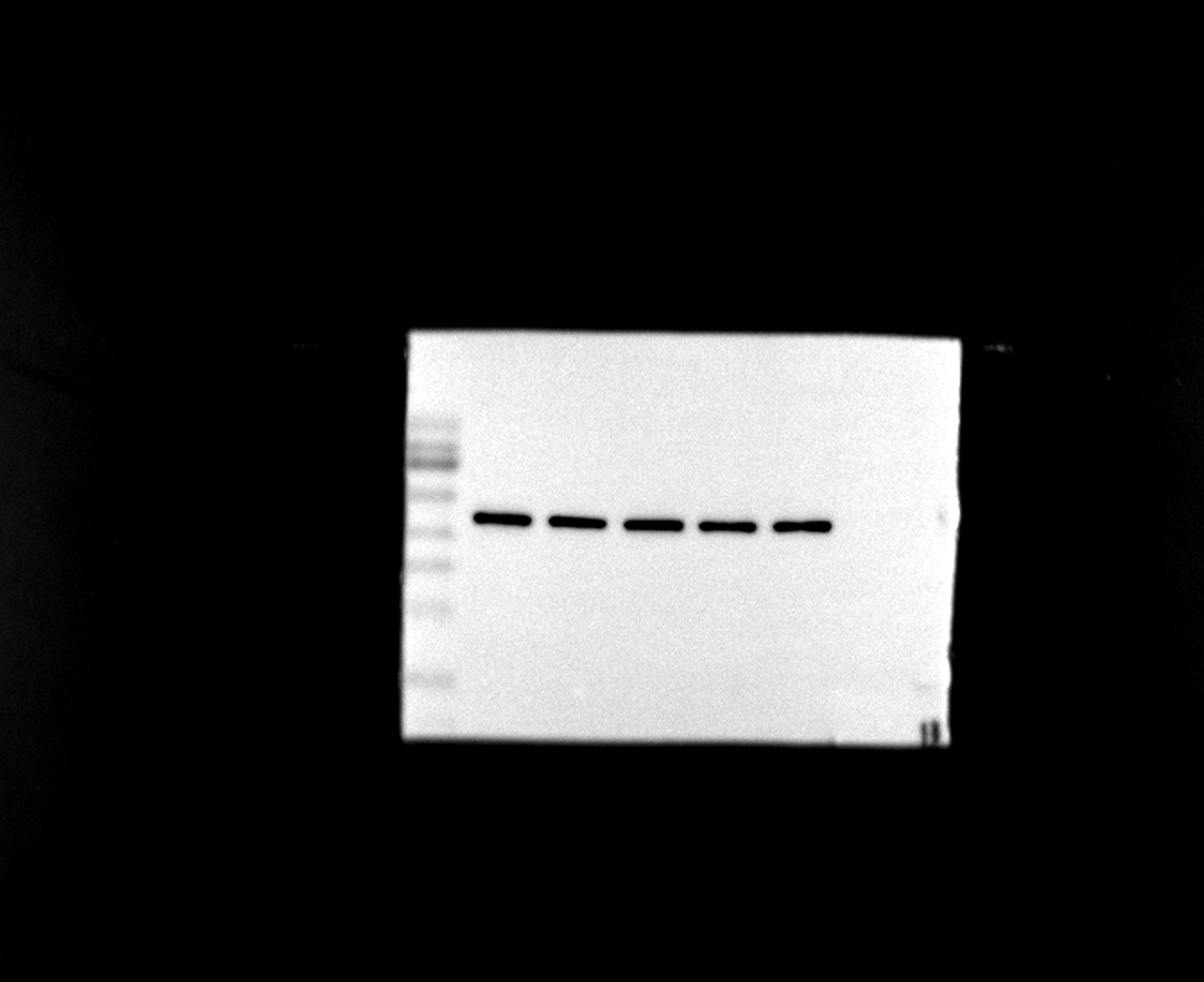

Supplement: Supplementary file 5 [file DataSheet1.zip › western blot images/β-actin-PC3.Tif]

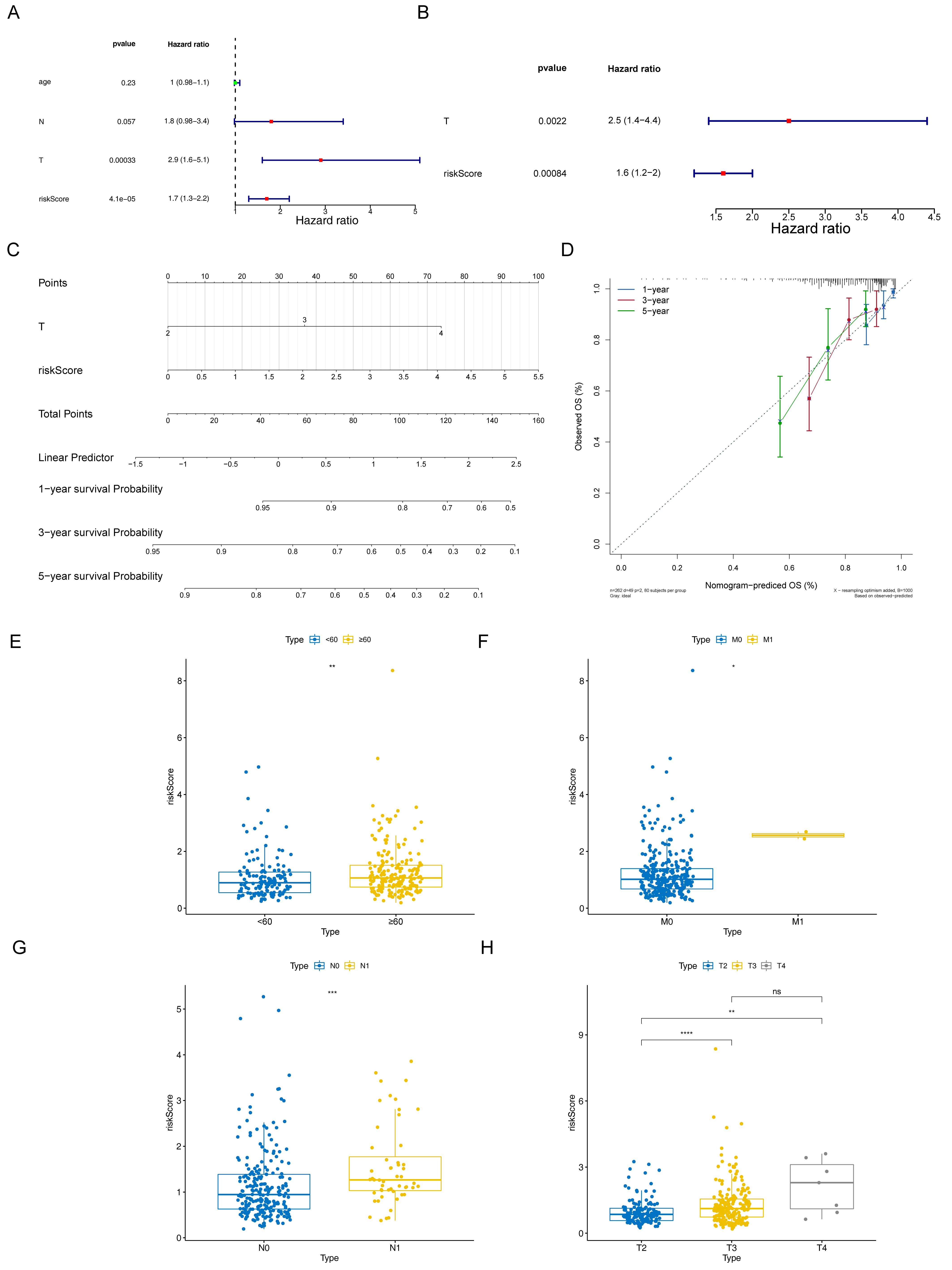

Supplement: Supplementary file 6 [file Image2.tif]

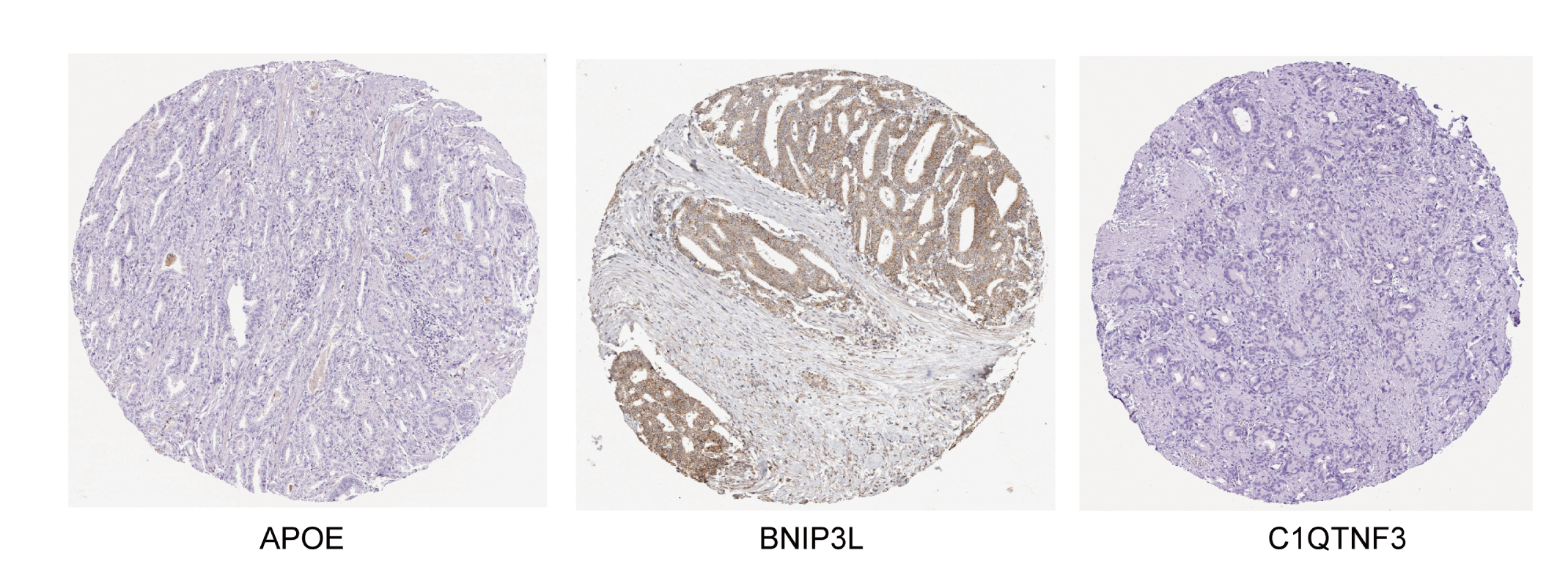

Supplement: Supplementary file 7 [file Image1.tif]
